# Rescue of a Live-Attenuated Porcine Epidemic Diarrhea Virus HSGP Strain Using a Virulent Strain and a Partially Attenuated Strain

**DOI:** 10.3390/v15071601

**Published:** 2023-07-21

**Authors:** Sok Song, Gyu-Nam Park, Jihye Shin, Ki-Sun Kim, Byung-Hyun An, SeEun Choe, Song-Yi Kim, Bang-Hun Hyun, Dong-Jun An

**Affiliations:** 1 Virus Disease Division, Animal and Plant Quarantine Agency, Gimcheon 39660, Gyeongsangbuk-do, Republic of Korea; ssoboro@korea.kr (S.S.); changep0418@korea.kr (G.-N.P.); shinji227@korea.kr (J.S.); kisunkim@korea.kr (K.-S.K.); ivvi59@korea.kr (S.C.); songkim@korea.kr (S.-Y.K.); hyunbh@korea.kr (B.-H.H.); 2 College of Veterinary Medicine, Seoul University, Gwanak-ro, Gwanak-gu, Seoul 08826, Republic of Korea; anbh5043@gmail.com

**Keywords:** PEDV, mutation, piglet, diarrhea, spike, deletion

## Abstract

In South Korea in 2013, the G1-based vaccine failed to prevent an outbreak of G2b-type porcine epidemic diarrhea virus (PEDV), which is more pathogenic than the traditional G1-type strain, thereby allowing the virus to spread. In 2017 and 2018, field samples were cultured sequentially on Vero cells to isolate HS (virulent) and SGP-M1 (partially attenuated) strains, respectively, of the G2b type. The HS strain harbors a single amino acid (aa) change and two aa deletions in the N-terminal domain of S1 (^55^I^56^G^57^E→^55^K^56^Δ^57^Δ). The SGP-M1 strain harbors a seven aa deletion in the C-terminal domain of S2 (^1380~1386^ΔFEKVHVQ). By co-infecting various animal cells with these two strains (HS and SGP-M1), we succeeded in cloning strain HSGP, which harbors the mutations present in the two original viruses. The CPE pattern of the HSGP strain was different from that of the HS and SGP-M1 strains, with higher viral titers. Studies in piglets showed attenuated pathogenicity of the HSGP strain, with no clinical symptoms or viral shedding, and histopathologic lesions similar to those in negative controls. These findings confirm that deletion of specific sequences from the S gene attenuates the pathogenicity of PEDV. In addition, HSGP strains created by combining two different strains have the potential for use as novel attenuated live vaccine candidates.

## 1. Introduction

Porcine epidemic diarrhea virus (PEDV), a single-stranded plus-sense RNA virus belonging to the alpha-coronavirus family, is an etiologic agent of porcine epidemic diarrhea (PED) [1,2]. PED causes severe acute watery diarrhea in piglets, leading to high morbidity and mortality, as well as huge economic losses to the global swine industry [3]. After an incubation period of 1–2 days, PEDV proliferates in the mucous epithelial cells of the small intestine of piglets and causes diarrhea [4,5,6,7,8,9]. Diarrhea results in the removal of the mucosal epithelial cells of the small intestine; even if the piglets consume milk, it is not absorbed in the intestine, causing more diarrhea [10,11]. PED viruses, which first emerged in England and Belgium at the end of the 1970s [1,12], are divided into groups 1 and 2 according to the type of gene they carry [13,14]. The G1 type is subdivided into G1a, which includes vaccine strains, and G1b, which primarily includes field strains [13,14]. The G2 type is subdivided into G2a and G2b [13,14]. Currently, it is the G2 type that causes diarrhea and mortality in many countries [4,6,7,9].

Eradication of circulating and persistent PEDV infections on pig farms will require a live vaccine, prepared by attenuating the G2b-type PEDV [15]. Typically, serial passage of viruses in a cell culture is performed to produce attenuated live vaccines; however, attenuation of the virus is not necessarily achieved simply through a large number of serial passages. Attenuated live vaccines are produced by passaging the virus several hundred times in host cells. However, this means that even if a mutation (such as a deletion) occurs in the genetic sequence of the virus, it still has to be tested in animals each time to see if it works. Thus, it can take a very long time to select a live vaccine candidate strain by checking for potentially pathogenic mutations in specific gene sequences of the virus. Therefore, unlike the traditional PEDV attenuation method, strategic attempts must be made to achieve the desired result with less time and effort. Based on the homology analysis of other coronavirus counterparts, the spike (S) protein of PEDV is divided into two domains, S1 (1–789 aa) and S2 (790–1383 aa) [16,17,18], each of which plays a role in mediating entry of PEDV into a host cell [19,20]. Specifically, the S1 domain is involved in receptor binding, and the S2 domain is involved in membrane fusion [19,20]. Thus, the receptor-binding capacity, and its role in viral entry, allows the S protein to determine PEDV invasion and release, tissue tropism, host range and interspecies spread, and even trypsin-dependent proliferation [17,20,21]. In addition, because the S protein stimulates production of neutralizing antibodies in the host [22], it is a primary target for the development of effective vaccines against PEDV. A previous study suggests that live-attenuated PEDV strains lack specific amino acids within the S1 and S2 domains [23].

To develop an attenuated live vaccine strain quickly, we established a single-clone attenuated live vaccine strain (HSGP) by co-infecting host cells with two gene-defective virus strains established by serial passage of wild-type PEDV and then mixed at a specific ratio. In addition, to confirm the feasibility of HSGP as a live-attenuated vaccine, it was administered to pigs via oral inoculation, followed by an assessment of viral proliferation and clinical symptoms, such as diarrhea and vomiting.

## 2. Materials and Methods

### 2.1. Cells and Viruses

Vero cells (ATCC^®^ CCL-81™, Manassas, VA, USA), MARC-145 cells (ATCC^®^ CRL-12231), and ST cells (ATCC^®^ CRL-1746™) were cultured at 37 °C/5% CO_2_ in DMEM supplemented with an antibiotic-antimycotic agent (Anti-Anti 100×, 5%) and fetal bovine serum (FBS 5%). The HS strain (genotype G2b) was isolated by emulsifying, filtering, and trypsinizing the small intestine of piglets with PED symptoms and residing on a pig farm in Hongseong-gun, Chungcheongnam-do (this farm experienced a PED outbreak in 2017). The SGP-M1 strain is a genotype G2b strain isolated from the small intestine and feces of piglets from a farm in Gimpo-si, Gyeonggi-do, in 2018.

### 2.2. Serial Passage of the HS and SGP-M1 Strains in Vero Cells

After growing Vero cells to 80–90% confluence in T75 culture flasks, the culture medium was discarded, and the cells were washed twice with phosphate-buffered saline (PBS, pH 7.2) to remove any remaining FBS components. Next, previously isolated HS and SGP-M1 strain stocks (1 mL of each) were mixed with 4 mL of infection medium (DMEM containing anti-anti (5%) and porcine trypsin (5 µg/mL)) and then dispensed into washed cell flasks. The flasks were incubated in a 37 °C/5% CO_2_ incubator for 1–2 h, with periodic shaking to allow the virus to fully penetrate the cells. The viral solution was then discarded, the cells were washed twice with PBS, and 15 mL of maintenance medium containing trypsin was added. When a cytopathic effect (CPE) was observed in 90% of the cells (after 1–2 days), the cells in a 75T flask containing 15 mL of medium were freeze (−70 °C)–thawed (37 °C) three times, and the solution was collected and centrifuged for 10 min at 3724× *g* to obtain a supernatant. The supernatant was then passed through a 0.45 µm syringe filter and used for the next passage.

### 2.3. Co-Infection of Cells with the HS and SGP-M1 Strains

The HS and SGP-M1 strains were diluted to a titer of 10^5.0^ TCID_50_/mL, and then mixed at the following ratios: 1:9, 3:7, 5:5, 7:3, and 9:1. The final inoculum volume for each group was adjusted to 1 mL, and Vero cells cultured in 6-well plates were then inoculated with the mixtures at a multiplicity of infection (MOI) of 1. When CPEs were observed at 2–3 days post-inoculation, the supernatant was harvested, and the cells were freeze–thawed three times. Conventional polymerase chain reaction (PCR) was performed using each of the harvested supernatants, followed by sequencing to confirm the chromatograms of each group. Next, stocks of inocula in which there was a high probability of co-existence of the two strains were prepared and then inoculated into cells (ST, Marc-145, and Vero) highly susceptible to infection by PEDV. Stocks harvested after inoculation onto each cell line were sequenced to select candidate groups for the final plaque assay.

### 2.4. Plaque Assay

Mixed virus stocks harvested from the co-infection experiments were inoculated into Vero cells seeded in 6-well plates. After 1 h, the virus solution was removed, and the cells were washed once. Then, Vero cells in each well were overlayered with 5 mL of agarose gel (a mixture of 3% agarose solution, 2× medium, antibiotic-antifungal solution, MEM NEAAS, and 2.5% trypsin), which was allowed to settle for 1 h at room temperature. After the gel had solidified, plates were incubated at 5% CO_2_/37 °C, and plaques were picked when CPEs appeared after 2 days. Plaque picking focused mainly on formation of syncytia with CPE morphology specific for the HSGP strain. Briefly, after identifying CPE under a microscope, the location of plaques showing CPE was marked on the plate. Next, a sterile micropipette tip was used to carefully pick individual plaques and transfer them to separating tubes containing cell culture medium. In addition, strains HS and SGP-M1 (obtained by plaque assay) were used for simultaneous infection.

### 2.5. Immunofluorescence Assay

The HS, SGP-M1, and HSGP strains were inoculated into 6-well plates seeded with Vero cells as described above. Once CPEs were confirmed, the inoculum was discarded, the plate was washed with PBS, and the cells were fixed in 80% acetone for 10 min at −20 °C. The acetone was discarded, and the cells were reacted for 1 h at 37 °C with a monoclonal antibody specific for the nucleocapsid (N) protein of PEDV (MEDIAN Diagnostics, cat no. RS-PED-11, Republic of Korea), followed by staining with a fluorescein-conjugated goat anti-mouse antibody. After washing, cell nuclei were stained with DAPI (4′,6-diamidino-2-phenylindole) (Abcam; catalog no. ab228549) at room temperature, followed by observation under a fluorescence microscope (200×).

### 2.6. Virus Growth Curve Analysis

Briefly, 96-well plates were seeded with Vero cells, followed by inoculation of HS, SGP-M1, and HSGP (each at 10^5.0^ TCID_50_/mL) at an MOI of 1. At 1 h post-infection, cells were washed prior to addition of infection medium (100 µL per well). Virus was then harvested vertically (8 wells, every 4 h up to 32 h). Titrations were performed in 96-well plates using viral stocks harvested at each time interval, and viral titers were measured using the Reed–Müench method. Data obtained from three repeat experiments were used for growth curve analysis.

### 2.7. Determination of the Pathogenicity of Each Strain in Piglets

To determine the suitability of the HSGP strain generated by co-infection of HS and SGP-M1 as a vaccine candidate, a pathogenicity attenuation test was conducted by oral administration to 5-day-old piglets born to sows that were not vaccinated against PEDV, porcine rotavirus (PoRV), or transmissible gastroenteritis virus (TGEV). These piglets were also negative for antibodies specific for diarrhea-causing viruses (PEDV, PoRV, and TGEV) in serum-neutralizing antibody tests. The titers of serum-neutralizing antibodies against PEDV, PoRV, and TGEV were determined in a virus neutralization test conducted in 96-well culture plates containing Vero, TF104, or ST cells. Briefly, the three cell lines (density = 2 × 10^4^ cells per well) were plated the day before the neutralization test. All test viruses (PEDV, PoRV, and TGEV) were diluted to 200 TCID_50_ in serum-free culture medium, mixed 1:1 with 2-fold diluted serum, and incubated at 37 °C for 1 h. Each of the three cell lines were inoculated with each mixture and incubated at 37 °C for 2 h. After removing the mixture, cells were washed twice with PBS and then maintained in virus growth medium at 37 °C in a 5% CO_2_ incubator. All neutralization titers were determined as the highest dilution of serum that inhibits virus-specific CPE. The experimental group was inoculated with a 30-passage HSGP strain, whereas the comparison groups received a 100-passage HS strain or a 100-passage SGP-M1 strain. Twenty piglets were divided into four groups (five piglets/group), three of which were orally administered the HSGP strain (final viral antigenicity, 1 × 10^5.0^ TCID_50_/dose), the HS strain (final viral antigenicity, 1 × 10^5.0^ TCID_50_/dose), or the SGP-M1 strain (final viral antigenicity, 1 × 10^5.0^ TCID_50_/dose). The remaining group was used as a negative control (NC). Piglets were observed clinically, weighed, and feces samples were collected for 6 days after oral administration. At 7 days post-inoculation, the piglets were autopsied, and feces and small intestine were collected for virus assessment and clinical observation of lesions, respectively. Diarrhea severity was determined according to a diarrhea score: 0 = normal (absence of diarrhea), 1 = soft (mild diarrhea), 2 = semi-fluid (medium-intensity diarrhea), 3 = watery diarrhea (severe diarrhea), and 4 = death. In addition, hematoxylin and eosin (H&E) staining was performed to confirm the histopathology of the small intestine (villous height and crypt depth).

### 2.8. Quantitative Assessment of PEDV Using Real-Time qPCR

An RNA extraction kit (RNeasy Mini Kit (Cat. No. 74104), Qiagen Inc., Maryland, MD, USA), a PCR premix kit (VDx PEDV qRT-PCR (Cat. No. NS-PED-31), Median Diagnostics Inc., Chuncheon, Republic of Korea), and a cDNA synthesis kit (HelixCript Easy cDNA Synthesis Kit (Cat. No. ECDNA100), Nanohelix Inc., Daejeon, Republic of Korea) were used for real-time qPCR. First, RNA was isolated from the feces or small intestine of each group of piglets using an RNA extraction kit, and cDNA was synthesized from the isolated RNA using a cDNA synthesis kit. The cDNA was then used in a PCR reaction under the following conditions: 50 °C for 30 min (1 cycle), 95 °C for 15 min (1 cycle), and 95 °C for 10 s/60 °C for 1 min (40 cycles). Positivity/negativity for PEDV, and PEDV replication, were determined based on the cycle threshold (Ct) value and the standard curve constructed using positive controls.

### 2.9. Statistical Analysis

All statistical analyses were performed using GraphPad Prism 6. Statistical significance was evaluated by one-way ANOVA method and statistical significance representations: * *p* < 0.05.

## 3. Results

### 3.1. Serial Passage of HS and SGP-M1

**Figure 1:**
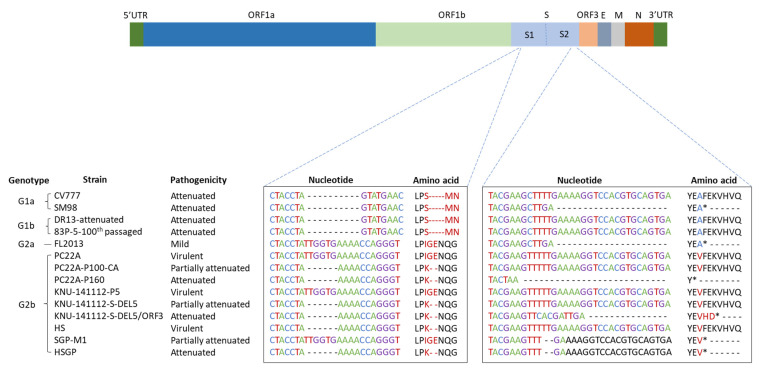
Comparison of nucleotide and amino acid sequences deleted from the virulent, partially attenuated, and attenuated strains. Two sites of the spike protein (aa 53–60 of the S1 protein and aa 1376–1386 of the S2 protein) were compared based on the USA/Colorado/2013 strain. The position of the deleted nucleotide and amino acid sequences is marked by dashes (---), and the termination codon is marked by an asterisk (*).

Isolation of wild-type PEDV in vitro revealed deletion of a six-nucleotide sequence, TTGGTG (164–169 bp), from the S1 domain of the S gene (Figure 1). Although the HS strain was cultured for over 100 generations, this six-nucleotide deletion remained. Thus, compared with the amino acid sequence of the standard PEDV strain (USA/Colorado/2013 (GenBank number: KF272920.1)) of the same genotype (G2b), the HS strain lacks amino acids glycine ‘G’ and glutamic acid ‘E’ at positions 55 and 56, respectively, in the S1 domain. Inoculation into Vero cells, followed by passage for up to 130 generations, did not introduce additional nucleotide mutations into the S1 gene of the HS strain. Unlike the HS strain, sequence changes in the S gene of the SGP-M1 strain were noted after passage 80. Compared with the nucleotide sequence of wild-type PEDV, we noted deletion of two nucleotides ‘TT’ from the 4136–4137 bp region of the S2 domain (Figure 1). This deletion results in a frameshift mutation in which the stop codon is pulled forward, thereby deleting seven amino acids from the S2 domain (phenylalanine ‘F’ at amino acid 1380; lysine ‘E’ at amino acid 1381; lysine ‘K’ at amino acid 1382; histamine ‘H’ at amino acid 1383; glutamic acid ‘E’ at amino acid 1381; lysine ‘K’ at amino acid 1382; valine ‘V’ at amino acid 1383; histidine ‘H’ at amino acid 1384; valine ‘V’ at amino acid 1385; and glutamine ‘Q’ at amino acid 1386). After that, even when the SGP-M1 strain was passaged up to 140 generations in vitro, no additional base mutations were introduced into the S gene.

### 3.2. Generation of HSGP Strains

**Figure 2:**
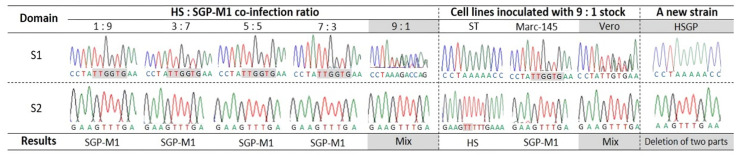
A novel attenuated PEDV HSGP strain was generated after co-infection with the HS and SGP-M1 strains. Changes in the nucleotide sequences of the S1 and S2 domains at various co-infection rates, and in three different cell types, are shown.

Vero cells were inoculated with the HS and SGP-M1 strains, mixed at a ratio of 1:9, 3:7, 5:5, 7:3, and 9:1, and virus genes were amplified from the collected supernatants by conventional PCR. The amplicons were then sequenced. The results showed that the SGP-M1 strains were detected in all groups apart from the group inoculated with the 9:1 mixture (Figure 2). However, the sequence chromatogram for the 9:1 group suggested co-existence of the two strains. Therefore, we selected single clones after passage of stocks from the 9:1 inoculum group. Briefly, the 9:1 stocks were used to infect Vero, ST, and Marc-145 cells, followed by PCR and sequence analysis (Figure 2). As a result, the virus infecting ST cells was identified as the HS strain, that infecting Marc-145 was identified as SGP-M1, and that infecting Vero cells was a mixture of both (Figure 2). Finally, supernatants taken from the Vero cell group were re-inoculated onto Vero cells and subjected to selective plaque picking over five generations, followed by PCR and sequencing. This resulted in the creation of a new strain with the characteristic deletions present in both HS and SGP-M1: this strain was named HSGP. In addition, the HSGP strain was identified as a G2b type in a comparative analysis of the nucleotide and amino acid sequences of several PEDV strains (Table 1). The ORF3 gene of the parental strains (HS and SGP-M1) and that of the newly created HSGP strain also showed 99.9% homology, with no genetic mutation.

### 3.3. Biological Characteristics of the HS, SGP, and HSGP Strains

**Figure 3:**
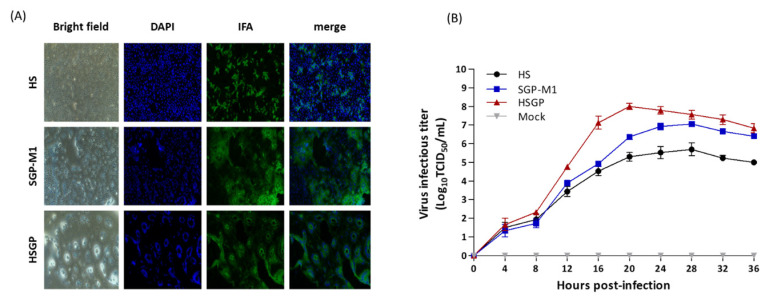
Cytopathic effects and time-dependent proliferation of Vero cells infected with the HS, SGP-M1, and HSGP strains. Bright field, DAPI, IFA, and merged images taken 3 days after the three strains were used to infect Vero cells (**A**). Changes in the virus infectious titer over time (**B**). HSGP, SGP-M1, HS, and Mock (negative control) are marked by red triangles, blue squares, black circles, and gray inverted triangles, respectively.

When Vero cells were inoculated with the HS, SGP-M1, and HSGP strains, all three showed a different pattern of CPEs. Upon inoculation with the HSGP strain, a large number of cell nuclei clustered in a circle, showing clear syncytium formation, whereas in the cells inoculated with the HS strain, the virus-infected cell nuclei were scattered. In cells infected with the SGP-M1 strain, atypical syncytium formation predominated, and scattered cell nuclei were also observed (Figure 3A).

The titers of the harvested viruses were measured over time, with the HSGP strain showing the highest titer at 20 h post-inoculation, and the HS and SGP-M1 strains showing the highest titer at 28 h post-inoculation. The highest viral titer for the HSGP strain was 10^8.0^ TCID_50_/mL, while that for the HS and SGP-M1 strains was 10^6.0^ TCID_50_/mL and 10^7.0^ TCID_50_/mL, respectively. These results indicate good cellular adaptability and a high proliferation rate of the HSGP strain, which was created by combining the HS and SGP-M1 strains (Figure 3B).

### 3.4. Clinical Signs in Suckling Piglets Infected with the HS, SGP-M1, and HSGP Strains

**Figure 4:**
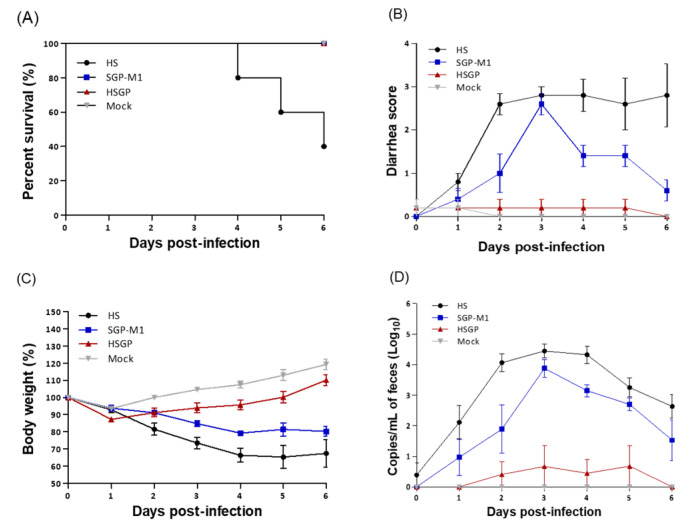
Pathogenicity of the HS, SGP-M1, and HSGP strains in suckling piglets. Survival rates (**A**), diarrhea scores (**B**), bodyweight changes (**C**), and PEDV RNA copy number in feces (**D**) after inoculation with the HS, SGP-M1, and HSGP strains.

As shown in Figure 4A, three piglets with particularly severe diarrhea (HS strain) died on Days 4, 5, and 6 after oral administration (60% mortality). As shown in Figure 4B, most pigs in the HSGP group did not have any diarrhea over the 6 days; two piglets had mild diarrhea (score of 1) at the beginning of the experiment. Considering that even in the NC group (non-inoculated group), mild diarrhea (score of 1 or less) appeared at 1 day post-inoculation (dpi), it is clear that the HSGP strain did not cause PEDV-associated diarrhea in piglets (Figure 4B). By contrast, the group of piglets that received the HS strain orally developed watery diarrhea (score of 3) for 2–6 days, similar to those infected with wild-type PEDV. Piglets treated orally with SGP-M1 had watery diarrhea (score of 3) up to dpi 3, but this improved to mild diarrhea (score of 1) after dpi 4, with no mortality. The weight gain/loss trends of 5-day-old piglets orally administered HS, SGP-M1, and HSGP strains were investigated. As shown in Figure 4C, a 10.15% weight gain at dpi 6 was observed in the piglet group that received the HSGP strain orally. The NC group (non-treated) had a weight gain of 19.32%. The HSGP group also gained weight, although not as much as the NC group. By contrast, the HS group piglets lost 32.54% of their bodyweight at dpi 6. Piglets in the SGP-M1 piglet group had lost 19.68% of their bodyweight by dpi 6. In other words, unlike the pigs that received the HSGP strain orally, those that received the HS and SGP-M1 viruses showed severe weight loss, which led to a decline in health (Figure 4C). We then compared the health of each group in terms of appetite and activity. The HSGP and NC groups had strong appetites and good activity levels during the experiment. However, the HS and SGP-M1 groups showed a significant decrease in activity after dpi 2–3, along with poor appetite and diarrhea and vomiting, all of which are typical symptoms of PEDV infection.

### 3.5. Virus Shedding in Feces and Viral Load in Different Intestinal Organs

After inoculating suckling piglets with the three strains, we measured the RNA copy number of PEDV in feces and intestinal tissue by real-time qPCR. As shown in Figure 4D, all three groups had the highest PEDV RNA copy number in feces at dpi 3 (NC: no PEDV RNA was detected). The amount of PEDV RNA in the feces of each experimental group was 5.22 (log_10_) for HS, 3.87 for SGP-M1, and 0.67 for HSGP, indicating that HSGP led to significantly less viral shedding than the other two viruses (Figure 4D). In addition, the PEDV RNA copy number in collected feces was very low after oral administration of the HSGP strain to piglets; no RNA was detected on the 6th day (Figure 4D). By contrast, feces collected from piglets in the HS group contained high RNA copy numbers on Days 3 and 4 after oral administration (Figure 4D).

Although fecal viral shedding in the SGP-M1 group decreased to a greater extent than that in the HS group, the RNA copy number was still around 2.42 (log_10_) at 6 dpi, significantly higher than that in the HSGP group (Figure 4D). After autopsy (at 7 dpi) of piglets orally inoculated with the HSGP, HS, and SGP-M1 strains, harvested intestines were emulsified and centrifuged, and RNA was extracted from the supernatant to determine the PEDV RNA copy number. As shown in Table 2, the RNA copy number in the duodenum, jejunum, ileum, and large intestine of the HSGP group was 1.66, 1.94, 2.56, and 1.66 (log_10_), respectively (negative control: no PEDV RNA was detected). By contrast, the RNA copy number was higher in the small and large intestine collected from piglets orally administered with the HS strain, ranging from a low of 2.52 (log_10_) to a high of 4.38 (log_10_) (Table 2). The RNA copy number in the small and large intestines from the SGP-M1 group ranged from 2.12 (log_10_) to 3.52 (log_10_) (Table 2). Common to all three strains, the highest amount of RNA was detected in the ileum (Table 2).

### 3.6. Gross and Histopathological Lesions in Suckling Piglets Infected with the HS, SGP-M1, and HSGP Strains

**Figure 5:**
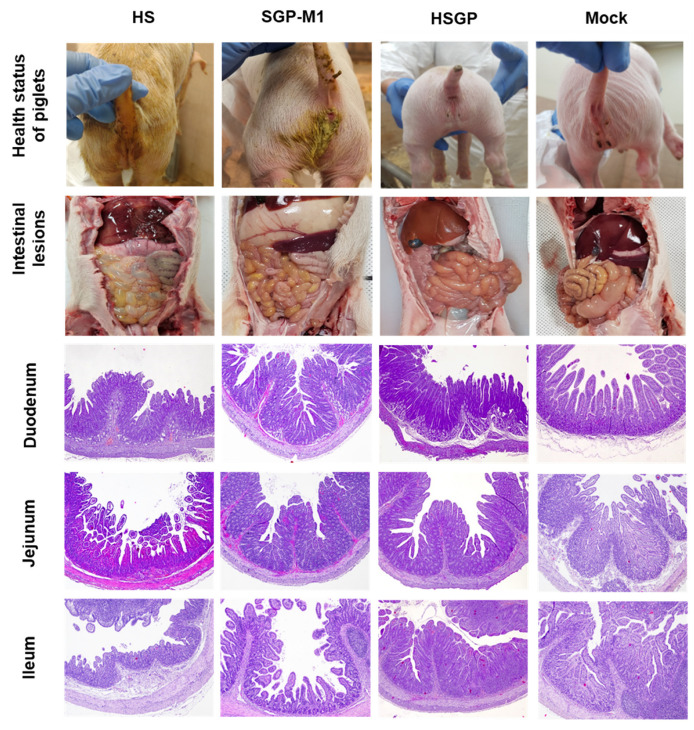
Health status, gross lesions, and changes in the morphology of the villi and crypts in the small intestine of suckling piglets inoculated with HS, SGP-M1, HSGP strains, and a negative control (Mock).

**Figure 6:**
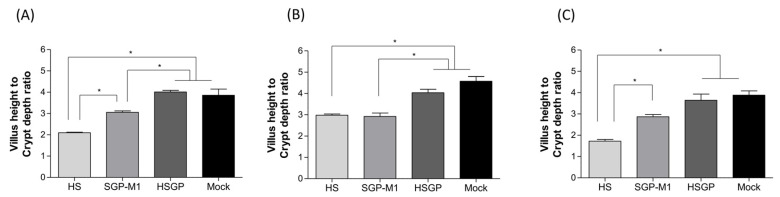
Villus height/crypt depth ratio in the small intestine of suckling piglets inoculated with strains HS, SGP-M1, and HSGP, or a negative control (Mock). Villus height/crypt depth ratio in the duodenum (**A**), jejunum (**B**), and ileum (**C**). The bar indicates the median. * *p* < 0.05 (comparison between each group).

All dead and euthanized piglets were autopsied, and pathological examination was performed. The autopsy indicated that the walls of the small intestine of all piglets infected with HS and SGP-M1 were transparent and thin, with HS showing the most severe changes. In some HS piglets, the lumen of the intestine was filled with a large amount of yellowish liquid. By contrast, no significant gross lesions were observed in the small and large intestines of the piglets in the HSGP group (Figure 5). Regarding histopathological lesions, the small intestines of the HS- and SGP-M1-infected groups showed mild to severe atrophy of intestinal villi compared with those of the HSGP and Mock groups (Figure 5). No significant histological intestinal lesions were observed in the HSGP piglets (Figure 5). Upon comparing the villous height:crypt depth (VH:CD) ratio among each experimental group, we observed that throughout the entire region of the small intestine (duodenum, jejunum, and ileum), the HS and SGP-M1 groups generally showed lower values than the HSGP and Mock groups (Figure 6). The lowest VH:CD ratio was observed in the HS group throughout the entire region of the small intestine (Figure 6). There was a statistically significant difference between the HS and SGP-M1 groups in the duodenum and ileum (*p* < 0.05) (Figure 6). By contrast, there was no significant difference between the HSGP and the Mock groups (Figure 6).

## 4. Discussion

Here, the HSGP strain was created by the multiple co-infection of cells with a virulent PEDV HS strain harboring a two aa deletion in the S1 domain (^56~57^ΔGE) and a partially attenuated PEDV SGP-M1 strain harboring a seven aa deletion in the S2 domain (^1380~1386^ΔFEKVHVQ). In addition, the S protein of the live-attenuated PEDV G2b-type oral vaccine (KNU-141112-S-DEL5/ORF3 strain) harbors a two aa deletion in S1 (^56~57^ΔGE) and a five aa deletion in S2 (^1382~1386^ΔKVHVQ) [24]. The HSGP strain showed the highest titer at 20 h after inoculation into Vero cells, and the CPE pattern was different from that of HS (virulent strain) and SGP-M1 (partially attenuated strain). We confirmed that virus replication was better than that of the highly pathogenic strain in Vero cells. The HSGP strain formed large syncytia in infected Vero cells, and there were differences in the CPE patterns induced by the HS and SGP-M1 strains. A previous study in which Vero cells were infected with an attenuated strain revealed larger syncytia and more virions, and a reduced number of S protein projections on the surface, compared with cells infected with the wild-type strain [25]. After inoculation of 5-day-old piglets with the HSGP strain, the survival rate was 100%, the diarrhea score was low, and there was almost no weight change. In addition, the PEDV RNA copy number in feces was very low. Typically, piglets infected with highly pathogenic PEDV show fecal viral RNA titers that peak at 1 dpi, or sometimes at 1–3 dpi; the titer declines markedly at the middle-to-late stages of infection and remains low during the recovery phase [4,6,8,26]. In infected gnotobiotic or normally lactating piglets (1–10 days old), a few (<5%) of the animals inoculated with highly pathogenic PEDV started to develop diarrhea at 1 dpi [4,5,6,7,8,9]. By contrast, a 3-day delay in the onset of diarrhea was observed in lactating pigs inoculated with S-INDEL PEDV [26,27].

Autopsy of piglets inoculated with the HSGP strain revealed little damage to the duodenum, ileum, and jejunum, and no changes to the villus height and crypt depth compared with controls, confirming that the HSGP strain was attenuated. However, piglets inoculated with the HS strain had particularly severe diarrhea, and three piglets died (60% mortality) by 6 dpi. In addition, high RNA copy numbers (10^4.5^ log10) were found in diarrhea feces on Day 3, and by Day 4 the piglets had lost about 30% of their body weight. The longer the observation period progresses, the higher the mortality caused by the HS strain is predicted to be, suggesting a highly pathogenic G2b type. The SGP-M1 strain, by contrast, was partially attenuated, resulting in no mortality but rather transient shedding of large amounts of virus by Days 3–5, and transient severe diarrhea on Day 3. The S1 domain of the PEDV S protein is responsible for binding to cell receptors, while the S2 domain is responsible for cell membrane fusion activity [19]. In the murine-adapted PEDV MK-p10 strain, loss of the ER retrieval signal KxHxx motif at the cytoplasmic tail of the S protein results in efficient transport of the S protein from the endoplasmic reticulum Golgi intermediate compartment to the cell surface, followed by increased fusion activity in vitro [19]. The nine aa deletion (EVFEKVHVQ), which includes an ER search motif, in PC22A-P120 leads to early termination of the S protein [23]. Similarly, PC22A-P120 shows higher cell fusion activity in vitro and lower toxicity in vivo than PC22A-P9 and PC22A-P100, suggesting that the nine aa (EVFEKVHVQ) deletion in S2 is associated with pathogenicity [23].

Previous studies compared the S genes of PEDV and tried to identify attenuated strains, partially attenuated strains, and virulent strains to select safer and more antigenically compatible attenuated live vaccine strains [23]. PEDV strain PN/Tottori2/2014, which harbors a large deletion in the S1 protein (194 aa deletion (residues 23–216)) [28], and US PEDV strain PC177 (197 aa deletion (residues 34–230)) [29] both show reduced virulence in pigs [28]. Currently, two major strains of PEDV are circulating in swine in the United States: a highly virulent non-S-INDEL strain and a less virulent S-INDEL strain [30]. Vaccination of pregnant sows with S-INDEL PEDV provides passive protection for piglets, thereby preventing viral replication and inducing an immune response in pregnant sows [30]. In addition, a comparative analysis of a pair of virulent and attenuated strains (PC22A, 83P5, DR13, YN, and CV777) revealed various mutation patterns related to different attenuations [23]. Serial passage of PC22A revealed that strain PC22A-P100C4 harbors a two aa (^56–57^ΔGE) deletion in the S1 protein, but has an intact S2 domain, and strain PC22A-P120 harbors a two aa deletion in the S1 region and a nine aa (^1378–1386^ΔEVFEKVHVQ) deletion in the C-terminal region of the S2 domain [23]. The PC22A-P100CA strain was partially attenuated for piglet inoculation, and the PC22A-P120 strain was supposed to be fully attenuated [23]. It was argued that several regions of the S protein of strain PC22A-P120 (e.g., the S1 domain and/or the C-terminus of S2) may affect the virulence of PEDV, while mutations in the ORF1ab, ORF3, E, and N genes may not [23]. In addition, strain PC22A-P160 harbors mutations in the ORF1ab (S221T in NSP2), S (D680N and F967L), and M (L150F) genes, in addition to aa deletions in the S1 and S2 domains (as in strain PC22A-P120). The authors reported that P160 further attenuated virulence [23] and claimed that strain PC22A-P160 was not pathogenic; rather, it induced active immunity, making it a candidate for an attenuated vaccine [23].

We found that the attenuated HSGP isolate could attain a higher infection titer than the HS and SGP-M1 strains of PEDV G2b in Vero cells. In the HSGP and SGP-M1 strains, the seven aa (FEKVHVQ) deletion in S2 results in premature termination of the S protein, presumably leading to increased cell fusion activity in vitro and reduced virulence in animals. Safety and attenuation confirmation tests in piglets revealed no diarrhea and very low levels of viral shedding in feces. Therefore, if the HSGP strain can replicate efficiently in inoculated pregnant sows and induce an appropriate immune response, it will be a potential candidate live-attenuated PEDV vaccine.

## 5. Conclusions

In conclusion, the HSGP strain created by deleting the S1 (^55^K^56^ΔG^57^ΔE) and S2 (^1380~1386^ΔFEKVHVQ) segments showed a high infection titer in Vero cells but did not cause diarrhea in piglets. In a future study, we will try to examine survival rates and protective ability further by inoculating piglets (born after intramuscular and oral inoculation of pregnant sows) with virulent PEDV. The results will determine whether the HSPG strain is a suitable attenuated vaccine candidate strain.

## Figures and Tables

**Table 1:** Homology of the nucleotide and deduced amino acid sequences of the complete spike gene of PEDV G1 and G2 genotypes.

Genotype		PEDV G1			PEDV G2		
	Strain	CV777	SM98	DR13 ^a^	PC22A ^b^	KNU14 ^c^	HSGP
PEDV G1	CV777	100 (100) ^d^	97.52 (96.14)	99.74 (99.88)	96.97 (93.11)	96.88 (93.44)	96.97 (93.52)
	SM98	-	100 (100)	97.42 (96.26)	96.44 (93.24)	96.51 (93.36)	96.47 (93.12)
	DR13	-	-	100 (100)	97.10 (93.18)	97.03 (93.52)	97.12 (93.59)
PEDV G2	PC22A	-	-	-	100 (100)	99.51 (99.47)	99.57 (98.98)
	KNU14	-	-	-	-	100 (100)	99.74 (99.15)
	HSGP	-	-	-	-	-	100 (100)

^a^ Live-attenuated PEDV DR13 strain. ^b^ PC22A-P160 strain. ^c^ KNU-141112-S-DEL2/ORF3. ^d^ Amino acid sequence (%).

**Table 2:** PEDV RNA copy number in the organs of piglets.

Organ	PEDV Strain		
	HS	SGP-M1	HSGP
Duodenum	2.52 ± 0.46 ^a^	2.14 ± 0.27	1.66 ± 0.33
Jejunum	3.22 ± 0.53	2.32 ± 0.46	1.94 ± 0.33
Ileum	4.38 ± 0.66	3.52 ± 0.39	2.56 ± 0.55
Large intestine	2.8 ± 0.51	2.12 ± 0.34	1.66 ± 0.23

^a^ Mean PEDV RNA copy number/g of tissue (log_10_) ± SD (standard deviation).

## Data Availability

The nucleotide sequences of the S gene of the HS, SGP-M1, and HSGP strains obtained in this study were submitted to the GenBank database under accession numbers OR045906-OR045908.
